# Editorial: Insights in bone research: 2023

**DOI:** 10.3389/fendo.2025.1570784

**Published:** 2025-02-25

**Authors:** Giacomina Brunetti, Jonathan H. Tobias

**Affiliations:** ^1^ Department of Biosciences, Biotechnologies, and Environment, University of Bari Aldo Moro, Bari, Italy; ^2^ University of Bristol, Musculoskeletal Research Unit, Bristol Medical School, Bristol, United Kingdom

**Keywords:** bone research, skeletal tissue, osteoporosis, endocrine disruptor, bone cells

Bone remodeling involves the coupled activity of osteoclasts and osteoblasts. The balanced activity of these cells leads to a healthy skeleton. Different factors can affect bone homeostasis and fracture risk, including inflammation, epigenetic modifications and dietary influences. This Research Topic summarizes a collection of papers published in the recent Research Topic “*Insights in Bone Research 2023*”, which together describe some of these influences.

Cassibba et al. aimed to investigate the relationship between denosumab treatment and coronavirus disease 2019 (COVID-19). The study included 331 osteoporotic patients treated with denosumab and 357 controls. The study shows that denosumab may be safely continued in COVID-19 patients, as it seems linked to a decreased incidence of symptomatic COVID-19, principally among the elderly. It has previously been demonstrated that denosumab may reduce the activity of pro-inflammatory cytokines, thus developing a potential immunomodulatory role (1). Consistent with this finding, the results of Cassibba et al. supported an interaction between RANKL antagonism and aging-associated inflammation.

Tang et al. evaluated the impact of the reduced bone mineral density (BMD) on traumatic rib fractures. The authors combined computed tomography (CT) and artificial intelligence (AI) to evaluate BMD and investigate its impact on traumatic rib fractures. The study involved 2,076 eligible patients, of whom 954 had normal BMD, 806 had osteopenia, and 316 showed osteoporosis. BMD screening based on CT-AI was suggested to be useful for the identification of subjects with reduced BMD, along with their fracture patterns.

Lui et al. reviewed the role of epigenetics in growth disorders. Epigenetic modifications play an important role in the regulation of transcription and gene expression. Epigenetic regulators include non-coding RNA, chromatin remodelers, and enzymes or proteins responsible for binding, reading, writing and erasing DNA and histone modifications. Recent discoveries in human genetics and high throughput sequencing technology have led to the identification of causative epigenetic regulators, for a wide variety of pediatric growth disorders, thus highlighting the role of potential epidrugs as a new type of therapeutic intervention.

Zheng et al. evaluated relationships between circulating inflammatory proteins and osteoporosis and fractures. In summary, the results suggested that CXCL11 may increase the risk of osteoporosis; IL-4, IL-7, IL-15RA, CXCL10, eotaxin/CCL11, and FGF23 may increase fracture risk; whereas IL-10RB could reduce fracture risk at the wrist and hand; CCL4 and MCP-3/CCL7 may decrease fracture risk of the femur, IFN-γ may reduce fracture risk of lumbar spine and pelvis; β-NGF and SIRT2 may decrease fracture risk.

Lerner et al. reviewed the role of Vitamin A on bone mass and fracture susceptibility. Vitamin A works as a hormone through nuclear receptors. It cannot be produced by the body, but it is supplied by the food as ß-carotene in fruits and vegetables and as retinyl esters in animal products. Its deficiency represents a risk factor for secondary osteoporosis and increased susceptibility to fractures. On the contrary, laboratory studies have found that high intake of vitamin A reduces cortical bone mass and makes bones weaker. Initial clinical studies reported contrasting results. Some authors found a negative association between vitamin A intake, bone mass and fracture susceptibility, whereas others observed no such associations. In clinical studies there is no consensus regarding the associations between serum retinol and BMD or hip fracture risk, thus further investigation is needed.

Botega et al. showed that refeeding partially reverses impaired fracture callus in undernourished rats. The study included (1) Sham: Sham rats with femoral fracture (2); FRes: Food-restricted rats with fracture (3), Fres+Ref: Fres rats with refeeding. Food restriction resulted in significant phenotypic changes in bone calluses when compared to sham rats characterized by deterioration in microstructure, reduced collagen deposition, BMD, and mechanical strength. Refeeding promoted bone callus collagen formation, decreased local resorption, and rescued the microstructural and mechanical changes caused by food restriction. Despite these benefits, bone callus density, OPG expression and collagen deposition are decreased with respect to shams. Thus, these authors concluded that food restriction had harmful effects on osseous healing, which was partially rescued by refeeding.

Chin reviewed the protective effects of tocotrienol (TT) on the skeleton and joints. TTs can be isolated from palm oil and annatto bean and showed effectiveness in improving joint and skeletal health in different animal models of osteoarthritis and bone loss, respectively. TT effects are mediated by anti-inflammatory, antioxidant, Wnt-suppressive, and mevalonate-modulating mechanisms in bone, as well as through self-repair mechanisms in chondrocytes. However, human clinical trials are limited. Thus, Chin concluded that TTs represent promising agents for the prevention of osteoporosis and osteoarthritis.

In conclusion, all these described mechanisms highlighted new potential targets for skeletal health.
